# Case Report: Fetal giant congenital hemangioma with arteriovenous malformation: to be or not to be

**DOI:** 10.3389/fmed.2026.1925509

**Published:** 2026-10-23

**Authors:** Peng Yue, Fan Ma, Li Ma, JiaoJiao Wan, Xiaoliang Liu, Fan Hu

**Affiliations:** 1 Key Laboratory of Birth Defects and Related Diseases of Women and Children of MOE, Department of Pediatrics, West China Second University Hospital, Sichuan University, Chengdu, China; 2 Department of Pediatrics, West China Second University Hospital-Tianfu·Sichuan Provincial Children’s Hospital, Meishan City, Sichuan Province, China

**Keywords:** congenital hemangioma, embolization coils, HAVF, pulmonary hypertension, sclerotherapy

## Abstract

**Objective:**

This study reports a rare and life-threatening case of a giant congenital hemangioma with arteriovenous fistula in a neonate and describes its clinical management and outcomes.

**Method:**

A female neonate with a giant right forearm hemangioma with arteriovenous fistula, detected prenatally at 23 weeks, was managed by a multidisciplinary team. Postnatally, the infant developed severe pulmonary hypertension and active bleeding from the mass. Diagnosis was confirmed via computed tomography angiography and arteriography. Emergency intervention with combined embolization coil and sclerotherapy was performed, followed by oral propranolol therapy.

**Results:**

Following the combined intervention and pharmacotherapy, the condition of the infant improved significantly. The severe pulmonary hypertension resolved, and the tumor size progressively reduced.

**Conclusion:**

Congenital hemangioma combined with arteriovenous malformation can lead to severe, life-threatening complications. A prompt, multidisciplinary approach involving embolization, sclerotherapy, and pharmacotherapy can achieve favorable outcomes, underscoring the importance of early recognition and integrated perinatal management.

## Introduction

Vascular lesions, including vascular tumors and malformations, are relatively common in neonates (1). Congenital hemangioma (CH) is a rare benign vascular tumor that is present at birth and fully developed. Based on the natural course, three main clinical subtypes have been identified: rapidly involuting congenital hemangioma (RICH), non-involuting congenital hemangioma (NICH), and partially involuting congenital hemangioma (PICH), whose characteristics and clinical behaviors overlap with those of the first two (2–4). There is growing evidence that at least most cases, whether they are RICH, NICH, or PICH, are due to somatic activating variants in GNAQ and its paralog GNA11 (5, 6). It indicates that the three belong to the same disease category rather than different diseases. CH can involve the head and neck, trunk, or limbs. It may cause fetal high-output heart failure, hydrops fetalis, anemia, thrombocytopenia, and death (7–13).

Vascular malformation is an abnormality in blood vessel morphogenesis. Unlike in tumors, endothelial cell renewal is normal in vascular malformations. According to the type of affected blood vessels and flow characteristics, vascular malformations can be classified as low-flow (capillary, venous, lymphatic, or mixed) or high-flow (arterial or arteriovenous) types. Arteriovenous malformation (AVM) is a high-flow type of vascular malformation, composed of abnormal arteries, veins, and capillaries. The direct arteriovenous communication leads to arteriovenous shunting (14). The common clinical manifestations of arteriovenous malformations in the trunk and extremities include pulsatile mass, pain, ulcer, bleeding, tissue necrosis, enlarged draining veins, venous hypertension, and/or heart failure. Due to its biological properties and associated high-flow shunting, AVM is more invasive. AVM is a relatively rare fast-flowing vascular malformation consisting of a complex network of primitive vessels directly connecting feeding arteries and draining veins. Complications due to these direct arteriovenous communications depend on their anatomical location and stage of development and include stroke, brain abscess, hypoxemia, or local rupture bleeding that may be life-threatening (15–17). Compared with other types of congenital vascular malformations, they are often accompanied by life- or limb-threatening complications. Therefore, early and active treatment of AVM is recommended (18–21). However, treatment options are limited, and AVM can cause significant morbidity and mortality (22).

Previous studies reported a rare case of a giant congenital hemangioma combined with an arteriovenous malformation, manifested with fetal malnutrition, fetal hydrops, and neonatal death (23). Another study reported an infant with a neonatal giant axillary hemangioma with a subclavian arteriovenous fistula (AVF) developed heart failure at birth (24). However, for those who can be born successfully, the gradual progress of the disease, as well as complications such as heart failure, hydrops fetalis, anemia, thrombocytopenia, etc., make the treatment particularly difficult, and it is necessary to cooperate with the multidisciplinary discussion to formulate a detailed diagnosis and treatment plan. Herein, we report a rare and life-threatening case of giant congenital hemangioma with arteriovenous fistula (HAVF), progressively leading to severe pulmonary hypertension and bleeding. Encouragingly, her clinical condition resolved after receiving the combination therapy of embolization and sclerotherapy, followed by oral propranolol.

## Case presentation

A fetus with a gestational age of 23 weeks, presenting with a progressively enlarging mass on the right forearm, was transferred to West China Second University Hospital. A multidisciplinary team comprehensively evaluates the condition of the fetus and closely follows up on the right limb mass. At 25^+3^ weeks of gestation, magnetic resonance imaging (MRI) findings revealed that the mass on the right forearm measured approximately 3.48 cm × 2.02 cm × 3.55 cm (Figure 1A, red arrows). It exhibited a multistrip expansion tube structure, and there was a possibility of hemorrhage (Figures 1A,B), indicating the possibility of congenital hemangioma. At a gestational age of 38 weeks, color Doppler ultrasound of the right upper limb showed mixed echo of the right forearm (6.1 cm × 7.4 cm × 8.2 cm), suggesting the possibility of vascular malformation. The fetus was born by cesarean section at 38^+2^ weeks of gestational age with a birth weight of 3,010 g and an Apgar score of 10-10-10. Physical examination revealed a mass measuring 7.0 cm × 6.5 cm × 5.0 cm on the right forearm of the infant, with a clear boundary and slightly high tension. The apparent dimensional differences between prenatal ultrasound and postnatal physical examination reflect methodological differences between *in utero* ultrasound measurement (which approximates the mass within a confined acoustic window) and postnatal caliper measurement on the body surface, rather than true tumor shrinkage. The surface skin was bluish-purple, part of the skin was broken and crusting, and the local skin temperature was slightly elevated. The infant showed no pain response (did not cry) on palpation of the mass, and skin edema was seen in the proximal and distal limbs compressed by the mass. The rest of the body was ruddy, without pallor, rash, or bleeding. The anterior fontanel was flat and soft. The physical examination of the heart, bilateral lungs, abdomen, and nervous system was negative. The vertical neck was fine, the muscle tone of the extremities was normal, and the right femoral artery was palpated with tremor.

**Figure 1:**
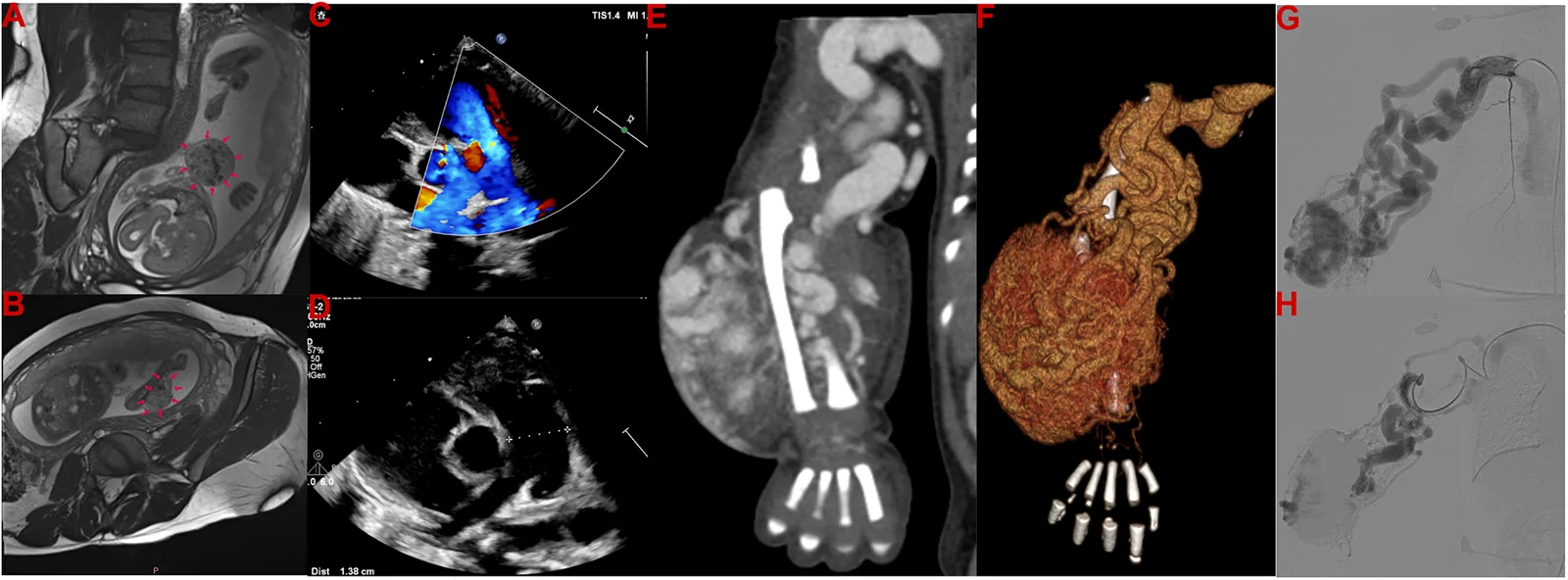
Imaging findings of fetal hemangioma with a giant arteriovenous fistula. **(A,B)** A fetus with a gestational age of 25^+3^ weeks, with MRI indicating a mass on the right upper with diamensions of 3.48 cm × 2.02 cm × 3.55 cm (red arrows), a multistrip expansion tube structure, and possibly bleeding. **(C,D)** Echocardiography revealing severe pulmonary hypertension (PG = 52 mmHg), accompanied by bidirectional shunt of the ductus arteriosus. **(E,F)** CTA indicating the presence of a hemangioma in combination with a giant arteriovenous fistula. **(G,H)** Arteriography confirming the arteriovenous fistula, and the emergency operation performed with the combination therapy comprising coil embolization and sclerotherapy.

Her condition deteriorated with the development of progressive dyspnea and bleeding from the mass two days after birth. Echocardiography disclosed severe pulmonary hypertension (estimated right ventricular systolic pressure = 52 mmHg, derived from tricuspid regurgitation peak velocity of 3.4 m/s using the modified Bernoulli equation [4V²+ estimated right atrial pressure of 5 mmHg]), accompanied by bidirectional shunt of the ductus arteriosus (Figures 1C,D). Chest radiography did not reveal any signs of neonatal respiratory distress syndrome, pneumonia, or pneumothorax. Computed tomography angiography (CTA) detected the presence of a giant congenital hemangioma in HAVF (Figures 1E,F). Serial laboratory monitoring was performed during the acute phase. On day 1, before the onset of bleeding, the platelet count was 182 × 10^9^/L, coagulation parameters (prothrombin time (PT) 12.4 s, activated partial thromboplastin time (APTT) 25.3 s, fibrinogen 2.86 g/L) were within normal limits, and D-dimer was markedly elevated at 6.67 mg/L (reference <0.5 mg/L), suggesting localized intravascular coagulation within the lesion. On day 2, coinciding with the active bleeding episode, the platelet count decreased to 73 × 10^9^/L, which is below the normal range but not profoundly thrombocytopenic; PT and APTT showed minimal prolongation (13.4 and 30.7 s, respectively), and fibrinogen fell slightly to 2.3 g/L, remaining within the normal range. D-dimer further increased to 8.15 mg/L, reflecting ongoing local fibrinolytic activity. On day 3, following emergency embolization and sclerotherapy, the platelet count recovered to 98 × 10^9^/L. Although the patient presented with active bleeding from the mass, Kasabach–Merritt phenomenon (KMP) was excluded for the following reasons. First, the platelet count (73 × 10^9^/L at its lowest) did not reach the profound thrombocytopenia (<50 × 10^9^/L) typically associated with classical KMP. Second, coagulation parameters (PT, APTT) remained near-normal with preserved fibrinogen (2.3 g/L), and the marked D-dimer elevation with normal fibrinogen suggested localized intravascular coagulation within the lesion rather than systemic consumptive coagulopathy. Third, the bleeding was localized to the site of skin ulceration rather than diffuse hemorrhage from multiple sites. Finally, KMP is most commonly associated with kaposiform hemangioendothelioma and tufted angioma and is exceedingly rare reported in congenital hemangioma. Therefore, the bleeding was attributed primarily to local ulceration and high-flow shunting rather than consumptive coagulopathy. Coagulation studies were not repeated postprocedure because clinical bleeding had ceased and the patient was hemodynamically stable.

Regarding progressive dyspnea, high-flow nasal ventilation was initiated. Moreover, our multidisciplinary team hypothesized that the progressive dyspnea might be associated with pulmonary hypertension, which might be possibly attributed to the giant hemangioma and arteriovenous fistula (23, 25). Therefore, arteriography further confirmed the arteriovenous fistula, and an emergency operation with a combination therapy consisting of coil embolization and sclerotherapy was immediately performed (Figures 1G,H). Emergency arteriography was performed via right femoral artery and vein puncture with placement of 5-Fr sheaths, after systemic heparinization (36.2 mg heparin sodium in 50 mL normal saline). Selective catheterization to the right subclavian artery using an FCO_2_ catheter confirmed a high-flow arteriovenous fistula with dilated, tortuous feeding arteries. A 4F Cobra catheter was then advanced into the dilated draining vein, and two 8-mm Nester coils (Cook Medical) were deployed. Subsequently, the skin over the lesion was prepared, and the lesional vessels were punctured at multiple points, through which eight additional Nester coils—three 10 mm, one 8 mm, three 6 mm, and one 4 mm—were deployed. In total, 10 Nester coils (4–10 mm) were placed. After all coils had been deployed, a mixture of 0.5 mL of iodized oil and 2 mL of absolute ethanol, followed by 3 mg of bleomycin plus 3 mL of contrast medium plus 1 mL of iodized oil, was injected for sclerotherapy. Post-procedural angiography demonstrated reduced lesion opacification and slowed venous drainage. The procedure was performed under general anesthesia with endotracheal intubation, and the infant was hemodynamically monitored throughout. No immediate procedural complications were observed, and the catheter and sheaths were removed with compression hemostasis. Serial hemoglobin levels remained stable throughout the acute phase. Despite local bleeding from the ulcerated mass, there was no evidence of clinically significant anemia, and no blood transfusion was required. At the same time, oral propranolol was prescribed at 1 mg/kg twice a day after excluding contraindications, and the dose was gradually increased to 2 mg/kg/day. During the titration period and for 72 h thereafter, continuous telemetry monitoring was maintained. Serial heart rate (every 2 h), blood pressure (every 4 h), and capillary blood glucose (every 4 h for 48 h, then every 6 h) remained within normal age-appropriate ranges, with no documented bradycardia, hypotension, or hypoglycemia. Encouragingly, her clinical condition finally improved, and the mass gradually reduced during the follow-up (Figures 2A,B). The infant achieved all age-appropriate developmental milestones, with a full range of motion, normal grip strength, and no residual edema or compression sequelae in the right forearm and hand.

**Figure 2:**
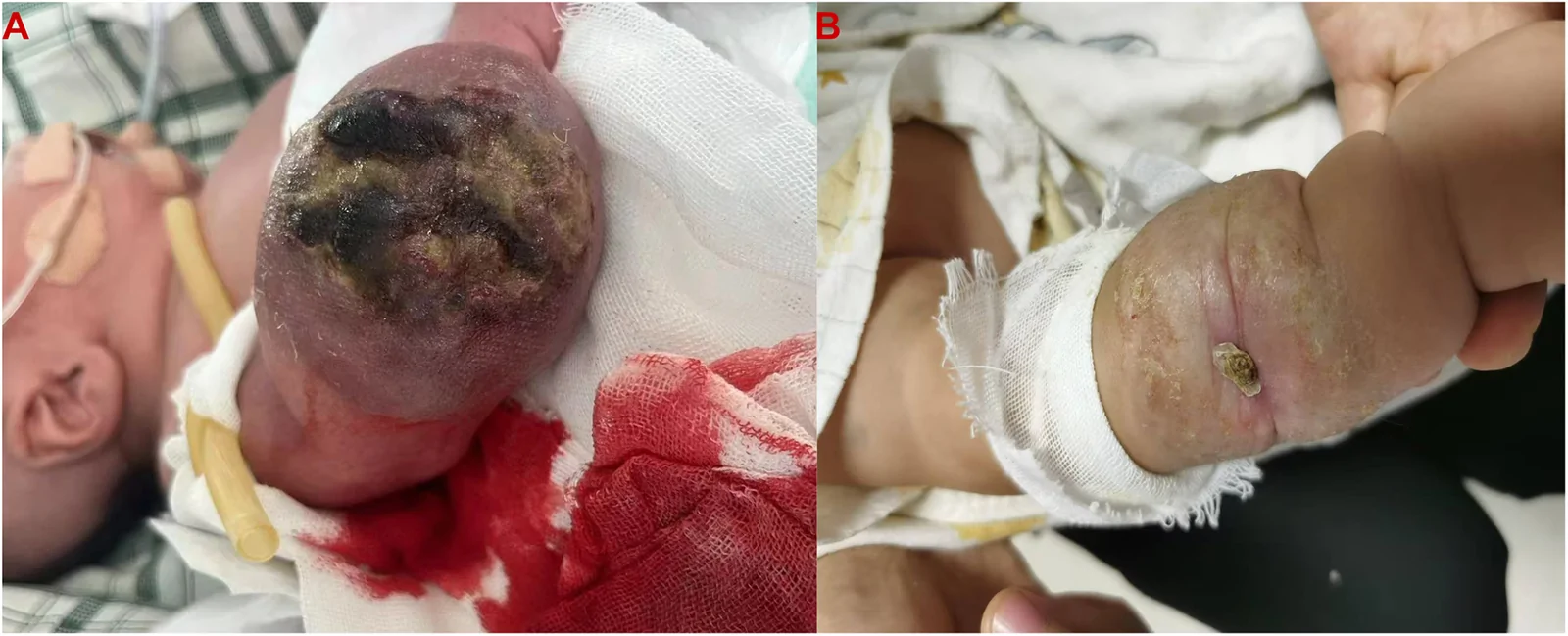
Clinical features of fetal hemangioma with a giant arteriovenous fistula. **(A)** After birth, the condition of the infant deteriorated with progressive dyspnea and the mass was bleeding. **(B)** The clinical condition of the infant finally improved.

Pre-treatment transthoracic echocardiography demonstrated moderate tricuspid regurgitation (peak velocity 3.5 m/s; peak gradient 49 mmHg), from which the pulmonary artery systolic pressure was estimated at approximately 52 mmHg, and trace pulmonary regurgitation (peak velocity 2.8 m/s; peak gradient 32 mmHg), from which the mean pulmonary artery pressure was estimated at approximately 35 mmHg (Figures 3A,B). There was also a 5.2-mm patent ductus arteriosus with predominantly right-to-left bidirectional low-velocity shunting, interatrial bidirectional shunting through two approximately 2-mm communications, right ventricular wall thickening (right ventricular wall thickening 3.1 mm), and D-shaped septal motion in the short-axis view, consistent with right ventricular pressure overload. Follow-up echocardiography (4 weeks after the baseline examination, 14 days after embolization) showed normal left ventricular systolic function (ejection fraction 66%, fractional shortening 34%) and normal right heart dimensions, with normal mitral and tricuspid inflow Doppler patterns (Figures 3C,D). No tricuspid regurgitation jet adequate for pulmonary artery pressure estimation was detectable, the ductus was no longer visualized, and only a small residual interatrial left-to-right shunt persisted, documenting complete resolution of the pulmonary hypertension.

**Figure 3:**
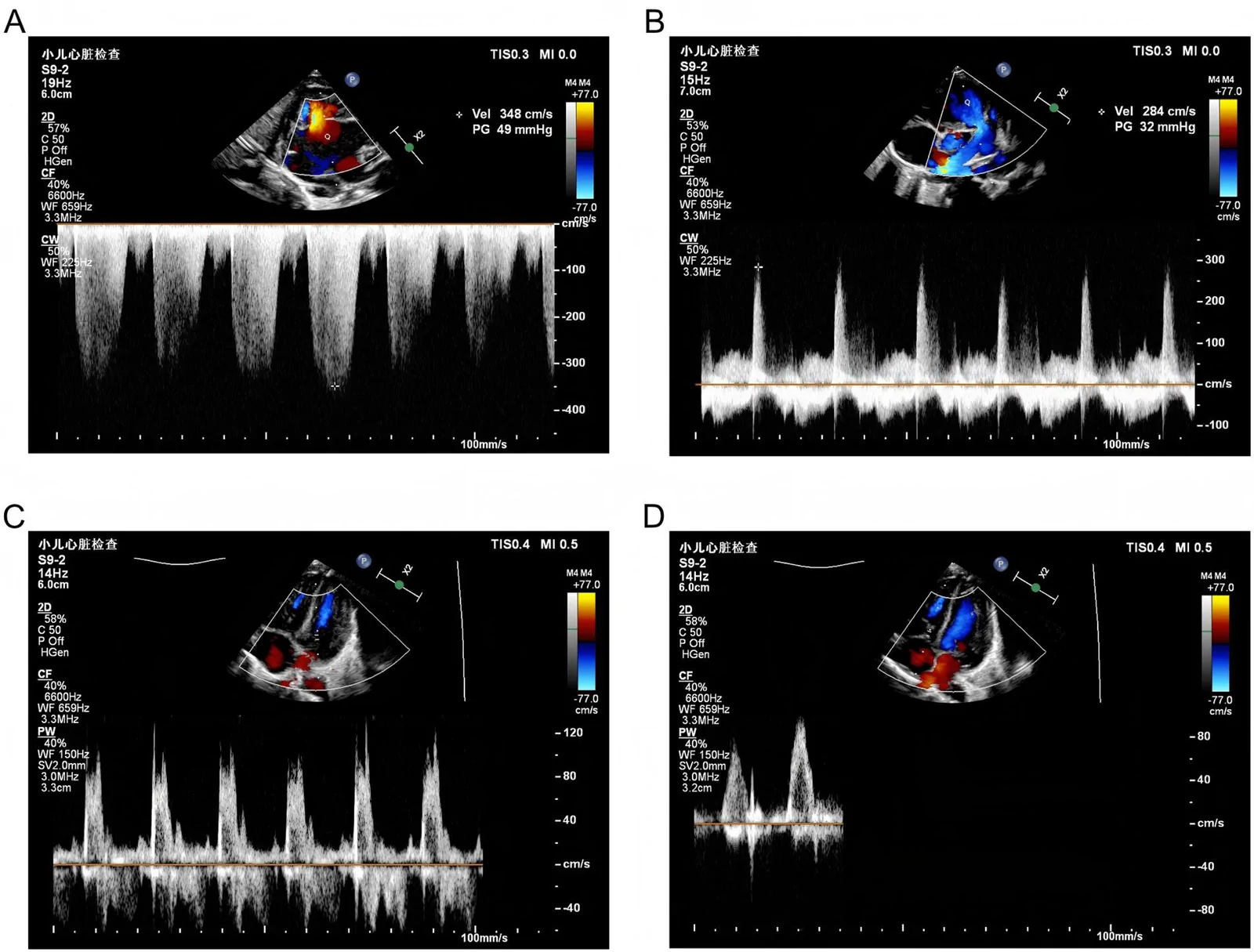
Transthoracic echocardiography documenting pulmonary hypertension before embolization and its resolution after treatment. **(A)** Pre-treatment apical four-chamber view with color Doppler demonstrating moderate tricuspid regurgitation; continuous-wave Doppler of the tricuspid regurgitant jet shows a peak velocity of 348 cm/s with a peak gradient of 49 mmHg, from which the pulmonary artery systolic pressure was estimated at approximately 52 mmHg. **(B)** Pre-treatment image showing trace pulmonary regurgitation; continuous-wave Doppler demonstrates a peak velocity of 284 cm/s with a peak gradient of 32 mmHg, from which the mean pulmonary artery pressure was estimated at approximately 35 mmHg. **(C)** Follow-up examination on 2 July 2024 (4 weeks after embolization): pulsed-wave Doppler of mitral inflow shows a normal inflow pattern with normal peak velocities, indicating unobstructed left ventricular filling. **(D)** Follow-up examination: pulsed-wave Doppler of tricuspid inflow shows normal low-velocity inflow; no tricuspid regurgitant jet sufficient for pulmonary artery pressure estimation was detectable, and no other echocardiographic sign of pulmonary hypertension was present, documenting resolution of the pulmonary hypertension.

## Discussion

In the clinical setting, giant congenital hemangiomas of the extremities combined with arteriovenous malformation have rarely been reported (23, 24). For the first time, we reported a rare and life-threatening giant congenital hemangioma accompanied by a giant arteriovenous fistula, which progressively led to severe pulmonary hypertension and bleeding. Encouragingly, her clinical condition resolved after receiving the combination therapy of coil embolization and sclerotherapy, followed by oral propranolol. Although parts of congenital hemangioma can regress without complications, it is likely congenital hemangioma (RICH) or NICH. The present study indicated that clinicians should be alert to the possibility that congenital hemangioma in combination with arteriovenous malformation might involve and present with life-threatening complications, such as severe pulmonary hypertension and bleeding.

Congenital hemangiomas are benign vascular tumors that differ from infantile hemangiomas in both biology and behavior (26, 27). Although infants with this disorder can resolve spontaneously without complications, parts of congenital hemangiomas are associated with life-threatening complications, such as ulceration, hypertrophic cardiomyopathy, heart failure, and pulmonary hypertension (23, 24, 27, 28), portal hypertension, and gastrointestinal bleeding (29, 30). Life-threatening complications were mostly found in infants with hepatic hemangiomas. These patients commonly had macrovascular shunting (arteriovenous, arterioportal, or portovenous) that decreased systemic blood volume and increased pulmonary blood volume, which further caused high-output cardiac failure. A review of the literature on giant congenital cutaneous hemangiomas revealed similar life-threatening complications, including congestive heart failure, hydrops fetalis, and coagulopathy, which have also been reported in well-documented cases (7–11, 23, 25, 31, 32). Liu et al. reported a neonatal giant axillary hemangioma with a subclavian arteriovenous fistula and heart failure, which was managed with respiratory support, improvement of myocardial contractility, blood transfusion, and promotion of lung maturation without arterial feeder embolization of the hemangioma in favor of an aggressive observation strategy (24). Liao et al. summarized the clinical features of 20 cases of congenital hemangioma. Among these fetuses, 70% (14/20) survived to birth, and their hemangiomas disappeared or regressed after receiving treatments with propranolol, interventional surgery, or were observed routinely (25). These complications were found in 40% fetuses, such as cardiac-related (88%)-elevated cardiothoracic area ratio, atrioventricular valve regurgitation, and fetal hydrops. The large hemangiomas (67% with maximum diameter ≥5 cm; 100% with a volume ≥40 cm^3^) were more likely to contribute to these complications, leading to advanced gestational age and a fast hemangioma growth rate (25).

Furthermore, in terms of pulmonary hypertension associated with congenital hemangioma and arteriovenous fistula, Mao et al. reported a female infant had congenital hepatic hemangioma with arteriovenous fistula, presenting with pulmonary hypertension, which was resolved after transcatheter arterial embolization (TAE); however, the recurrence of hepatic hemangioma required oral propranolol therapy, which led to complete regression by 18 months of age (33). It is widely recognized that HAVF is a rare congenital vascular malformation associated with high mortality, associated with severe pulmonary hypertension, right atrial and ventricular enlargement, and postnatal tachycardia (34). Among HAVF patients, more than 50% develop heart dysfunction, with 50%–90% dying of severe heart failure within the first week of life (34). Moreover, it includes three types: hepatic artery-to-portal vein, hepatic artery-to-hepatic vein, and portal vein-to-hepatic vein fistulas, which present with different clinical manifestations (29, 35). Multiple abnormal arteriovenous shunts between the hepatic artery, portal vein, and hepatic vein, combined with a large hepatic hemangioma, led to increased right cardiac load and pulmonary congestion. In the present study, we reported a rare case of a congenital hemangioma and arteriovenous fistula located in the infant's right forearm, presenting with severe pulmonary hypertension and bleeding. It indicated that HAVF involving the limbs also developed the life-threatening complications, severe pulmonary hypertension and bleeding, which can be resolved by the combination therapy of coil embolization, sclerotherapy, and propranolol. Moreover, our study suggested that a multidisciplinary management was crucial for this disorder during the perinatal stage.

In terms of managing HAVF, prompt diagnosis is the cornerstone. Clinically, the integration of clinical features with imaging ﬁndings supported the diagnosis. First, clinicians should choose tools such as ultrasound, CT, and angiography as essential diagnostic modalities. Moreover, regarding hemangiomas and vascular malformations occurring during the fetal period, multidisciplinary management is required to comprehensively evaluate the clinical features, especially their evolution. Second, clinicians should identify the risk factor of life-threatening complications among patients with congenital hemangioma and arteriovenous fistula. Regrettably, previous studies have only found that the volume of hemangiomas (maximum diameter ≥5 cm; a volume ≥40 cm^3^) indicates a possible risk factor for these complications (25). Prognostic variability in these benign tumors correlates with tumor size, growth rate, and intralesional shunting, manifesting clinically from asymptomatic to life-threatening presentations (36). Further studies should be carried out to identify these risk factors for facilitating the clinical management of patients with HAVF who have life-threatening complications.

Based on the clinical condition of patients with HAVF, clinicians should implement different therapeutic approaches. Notably, current therapeutic strategies include aggressive observation, interventional procedures, pharmacotherapy, and even surgical resection. Neonates present with poor tolerance and high risks of hemorrhage-related complications; therefore, it is not recommended to perform surgical resection as the initial treatment for neonates with congestive heart failure. Compared with surgical intervention, transcatheter arterial embolization has more advantages: it can rapidly block arteriovenous shunting while alleviating symptoms and reducing perioperative stress, with the minimally invasive nature (30, 37). However, neonates received transcatheter arterial embolization, which remains technically challenging, especially for patients with congenital hepatic hemangiomas and arteriovenous fistulae (34, 37). In addition, propranolol, as another important therapeutic agent, can reduce lesion size by vasoconstriction and by inhibiting the formation of vascular endothelial growth factor and fibroblast growth factor, as well as cell proliferation (36, 38, 39).

The decision to pursue emergency embolization and sclerotherapy in our case, rather than adopting an observational strategy, warrants comparison with a previously reported case. Liu et al. described a premature neonate with a giant axillary hemangioma and subclavian arteriovenous fistula complicated by high-output heart failure, which was successfully managed with an “aggressive observation strategy” after the infant showed gradual clinical improvement (24). However, several key differences justified our interventional approach. First, our patient presented with active bleeding from an ulcerated mass, which posed an immediate risk of hemodynamic instability. Second, the lesion was substantially larger (maximum diameter 8.2 vs. 4.5 cm) and associated with severe pulmonary hypertension (pressure gradient = 52 mmHg) that progressed despite respiratory support. Third, the clinical trajectory was one of rapid deterioration rather than gradual improvement. In the context of life-threatening complications, active hemorrhage and progressive pulmonary hypertension with failure of conservative measures, prompt intervention was deemed necessary. This contrasts with the more indolent course described by Liu et al., where the condition of the infant improved with medical support alone, allowing embolization to be deferred. These observations suggest that the decision to intervene vs. observe in neonates with giant congenital hemangioma and arteriovenous fistula should be guided by the severity and trajectory of clinical complications, with intervention reserved for cases complicated by active bleeding, progressive hemodynamic compromise, or failure of conservative management.

We acknowledge a major limitation of this case: the diagnosis of congenital HAVF was based solely on prenatal and postnatal imaging, including MRI, Doppler ultrasound, CTA, and angiography. Histopathological examination and molecular testing for somatic GNAQ/GNA11 mutations, which are characteristics of CH, were not performed. This was primarily due to the unstable clinical condition of the neonate and the high risk of bleeding from the mass, which precluded safe biopsy. Therefore, while the imaging findings (a large, heterogeneous mass with high-flow shunting) are highly suggestive of CH with AVM, we cannot definitively exclude the possibility of a primary high-flow arteriovenous malformation with secondary mass effect or soft-tissue changes. The distinction between these entities remains challenging without tissue confirmation, and our diagnostic conclusion should be interpreted with caution. Future cases should aim for genetic or histopathological correlation whenever clinically feasible.

The use of propranolol in this case requires careful justification, particularly given the pathobiological distinction between CH and infantile hemangioma (IH). Unlike IH, which is GLUT1-positive and responds to beta-blockers through β-adrenergic-mediated vasoconstriction and inhibition of angiogenesis, CH is driven by somatic activating mutations in GNAQ/GNA11, and its response to propranolol is not well established. Indeed, Wang and Guo recently criticized the use of oral propranolol for hepatic CH, arguing that beta-blockers are only used to treat infantile hemangioma in current evidence-based literature (40). While some case reports have described successful outcomes with propranolol in CH (41), there is no consensus, and evidence remains limited to anecdotal reports. In our case, propranolol was administered as an off-label adjunctive therapy following emergency embolization and sclerotherapy, based on a multidisciplinary risk–benefit assessment in a life-threatening situation. We acknowledge that this single favorable outcome does not establish efficacy, and we emphasize the need for further research to determine whether subsets of CH patients may benefit from beta-blocker therapy.

## Conclusion

Clinicians should be aware that congenital hemangioma in combination with arteriovenous malformation might present with life-threatening complications, such as severe pulmonary hypertension and bleeding. However, patients with this disorder might benefit from prompt management, such as the transcatheter arterial embolization, sclerotherapy, and propranolol, which were provided by a multidisciplinary team during the perinatal stage.

## Data Availability

The original contributions presented in the study are included in the article/Supplementary Material; further inquiries can be directed to the corresponding authors.
